# Preventing Paradoxical Tuberculosis-Associated Immune Reconstitution Inflammatory Syndrome in High-Risk Patients: Protocol of a Randomized Placebo-Controlled Trial of Prednisone (PredART Trial)

**DOI:** 10.2196/resprot.6046

**Published:** 2016-08-29

**Authors:** Cari Stek, Charlotte Schutz, Lisette Blumenthal, Friedrich Thienemann, Jozefien Buyze, Christiana Nöstlinger, Raffaella Ravinetto, Edwin Wouters, Robert Colebunders, Gary Maartens, Robert J Wilkinson, Lutgarde Lynen, Graeme Meintjes

**Affiliations:** ^1^ Institute of Infectious Disease and Molecular Medicine Clinical Infectious Diseases Research Initiative University of Cape Town Cape Town South Africa; ^2^ Institute of Tropical Medicine Department of Clinical Sciences Antwerp Belgium; ^3^ Department of Medicine University of Cape Town Cape Town South Africa; ^4^ Institute of Tropical Medicine Department of Public Health Antwerp Belgium; ^5^ Department of Sociology Centre for Longitudinal and Life Course Studies University of Antwerp Antwerp Belgium; ^6^ Department of Medicine Imperial College London London United Kingdom; ^7^ The Francis Crick Institute Mill Hill Laboratory London United Kingdom

**Keywords:** tuberculosis, HIV, paradoxical TB-associated immune reconstitution inflammatory syndrome (TB-IRIS), corticosteroids, prednisone, randomized controlled trial

## Abstract

**Background:**

Early antiretroviral therapy (ART) initiation in patients diagnosed with HIV-associated tuberculosis (TB) reduces mortality among those with the lowest CD4 counts. At the same time, both early ART and a low CD4 count heighten the risk of paradoxical TB-associated immune reconstitution inflammatory syndrome (TB-IRIS). TB is common in patients starting ART in sub-Saharan Africa. Safe interventions that reduce the incidence or severity of TB-IRIS are needed. Prednisone has been shown to reduce symptoms and markers of inflammation when used to treat TB-IRIS.

**Objective:**

To determine whether prophylactic prednisone in patients at high risk for paradoxical TB-IRIS initiating ART reduces the incidence of TB-IRIS.

**Methods:**

We are conducting a randomized, double-blind, placebo-controlled trial of prophylactic prednisone (40 mg/day for 2 weeks, followed by 20 mg/day for 2 weeks) initiated at the same time as ART in patients at high risk for TB-IRIS (starting ART within 30 days of TB treatment and CD4 count **≤**100/μL). The primary endpoint is development of TB-IRIS, defined using an international consensus case definition. Secondary endpoints include time to TB-IRIS event, severity of TB-IRIS, quality of life, mortality, hospitalization, other infections and malignancies, and adverse events including corticosteroid adverse effects.

**Results:**

Enrollment for the trial began in August 2013. All 240 participants have been enrolled, and safety follow-up will be completed in March 2017.

**Conclusion:**

No preventive strategies for TB-IRIS currently exist. If results of this trial demonstrate the efficacy and safety of prednisone, this will provide clinicians with an evidence-based preventive strategy in patients at high risk for paradoxical TB-IRIS when initiating ART.

## Introduction

### Overview

Tuberculosis (TB) is the most common opportunistic disease affecting HIV-1-infected patients in low- and middle-income countries; up to 42% of patients starting antiretroviral therapy (ART) in sub-Saharan Africa are on treatment for active TB [1]. When ART is commenced in patients on treatment for active TB, an immunopathological reaction known as paradoxical TB-associated immune reconstitution inflammatory syndrome (TB-IRIS) is reported in 18% of cases (95% CI 16%-21%), resulting in new or recurrent TB-related signs and symptoms. The most common clinical features are (1) pulmonary features such as recurrent cough, chest pain, and worsening radiographic pulmonary infiltrates and (2) inflammation and enlargment of lymph nodes. Although mortality attributed to TB-IRIS is relatively low (2%, 95% CI 1%-3%), TB-IRIS causes considerable morbidity, with 25% (95% CI 19%-30%) of cases requiring hospitalization [2]. Neurological involvement, less frequent than pulmonary and nodal involvement, is associated with substantial mortality [3].

Several clinical trials have evaluated the optimal timing of ART initiation in ART-naïve HIV-infected patients diagnosed with active TB. These were included in a recent meta-analysis which showed that early ART (around 2 weeks into TB treatment) in patients with CD4 counts **≤**50/μL improved survival compared with starting at around 8 weeks [4]. However, low CD4 count and shorter interval between TB treatment and ART are two of the most important risk factors for TB-IRIS [2]. The same meta-analysis showed that early ART more than doubled the risk of TB-IRIS. In patients with a CD4 count <50/μL, the risk of TB-IRIS with early ART is particularly high [5,6]. Thus, while international guidelines now advise ART within 2 weeks in TB patients with CD4 <50/μL, it can be anticipated that this will increase the risk of TB-IRIS. Despite this, no evidence-based strategy for preventing TB-IRIS currently exists. TB-IRIS will continue to be a major complicating factor in ART programs in sub-Saharan Africa, even with the new World Health Organization guidelines recommending to start ART in all newly diagnosed HIV-infected patients regardless of CD4 count [7], because many HIV-infected individuals still enter care with low CD4 counts and active TB [8-10]. Therefore, interventions to reduce the incidence of TB-IRIS are urgently needed. TB-IRIS is thought to result from an exaggerated immune response in the context of rapidly recovering immunity in the presence of abundant *Mycobacterium tuberculosis* antigen at sites of disease. Attenuating this aberrant inflammatory response during early ART with corticosteroids may prevent TB-IRIS or at least reduce the severity of TB-IRIS clinical manifestations.

### Corticosteroids in the Treatment of Tuberculosis

The host immune response contributes to pathology caused by TB [11], and corticosteroids have been used as adjunctive treatment in TB for several decades [12]. Corticosteroid treatment does not diminish the efficacy of TB treatment [12]. Evidence of significant clinical benefit from controlled clinical trials exists for treatment of TB meningitis, where it reduced short- to medium-term mortality [13,14], and pericardial TB, where it reduced the complication of constriction [15], as well as in the treatment of paradoxical TB-IRIS [16].

A Cochrane systematic review of corticosteroids as an adjunct to TB treatment in TB meningitis showed that corticosteroids reduced the risk of death (relative risk 0.78, 95% CI 0.67-0.91). The survival benefit occurred irrespective of the severity of TB meningitis [13]. One of the studies included in the review also demonstrated significantly fewer severe adverse events in patients who received dexamethasone [17]. In particular, eight cases of severe drug-induced hepatitis occurred in the placebo group and none in the dexamethasone group. Adverse drug reactions are another major complicating factor in the management of HIV-associated TB. By reducing the incidence of these hypersensitivity reactions, corticosteroids could potentially reduce morbidity, mortality, and the burden on limited health care resources.

The Investigation of the Management of Pericarditis (IMPI) trial was conducted in several African countries and evaluated prednisolone (and *M indicus pranii* in a factorial design) in 1400 patients with tuberculous pericarditis, of whom 67% were HIV-infected [15]. Participants were assigned to receive either prednisolone or placebo for a period of 6 weeks. The primary outcome was a composite of death, the first occurrence of cardiac tamponade requiring pericardiocentesis, or constrictive pericarditis. There was no significant difference in the primary outcome between patients who received prednisolone and those who received placebo. Prednisolone therapy, however, was associated with significant reductions in the incidence of constrictive pericarditis and hospitalization, which were secondary endpoints.

In the IMPI trial, prednisolone was associated with an increase in cancers (1.8% vs 0.6%, *P*=.03). These were mainly HIV-related cancers, and most patients who developed HIV cancers were not taking ART at the time of enrollment to the trial (personal communication, IMPI investigators). These findings added to concerns regarding the use of adjuvant corticosteroids in HIV-infected patients present from two prior trials conducted in Uganda evaluating prednisolone in HIV-associated TB, both conducted prior to ART availability. One showed that prednisolone was associated with more rapid clinical and radiological improvement but also with an excess of Kaposi sarcoma [18]; the other showed more rapid clearance of *M tuberculosis* from sputum with prednisolone treatment but also a transient increase in HIV viral load (as well as worsening of underlying hypertension, fluid retention, and hyperglycemia) [19]. A third trial in Zambia showed more reactivation of herpes zoster during prednisolone use in HIV-associated TB [20].

### Corticosteroids in the Treatment of Tuberculosis-Associated Immune Reconstitution Inflammatory Syndrome

We previously conducted a randomized double-blind placebo-controlled trial of prednisone for the treatment of paradoxical TB-IRIS [16]. A 4-week course of prednisone (1.5 mg/kg/day for 2 weeks followed by 0.75 mg/kg/day for 2 weeks) resulted in significantly reduced hospitalization and need for outpatient therapeutic procedures, and more rapid clinical improvement. There was no excess of corticosteroid metabolic adverse effects or severe infections in the prednisone arm. Significant decreases in the serum concentrations of interleukin 6, interleukin 10, interleukin 12p40, tumor necrosis factor α, interferon γ, and interferon γ-inducible protein 10 on prednisone suggest that the beneficial effect of corticosteroids in TB-IRIS was mediated at least in part through the attenuation of proinflammatory cytokine responses [21]. We have previously demonstrated that these same cytokines are differentially increased in the serum of HIV-associated TB patients who develop TB-IRIS compared to control subjects who start ART and do not [22].

In HIV-infected patients with pneumocystis pneumonia, early adjunctive treatment with corticosteroids reduces mortality and the risk of mechanical ventilation. It is presumed that as organisms are killed, inflammation increases in the lungs, leading to a worsening of the patient’s clinical condition; corticosteroids possibly reduce or prevent this worsening by reducing inflammation [23].

We are currently conducting a randomized controlled trial (the PredART trial) in Khayelitsha, South Africa, with the primary objective to determine whether a 4-week course of prednisone in patients at high risk for TB-IRIS (starting ART within 30 days of starting treatment for TB and a CD4 count of ≤100/μL) reduces the incidence of TB-IRIS. This PredART trial is different to our prior treatment trial [16] in that prednisone is being evaluated as a preventive rather than treatment strategy and given at lower doses.

Among the secondary objectives, we aim to determine whether prednisone reduces the severity of TB-IRIS and the risk of hypersensitivity drug reactions and consequent drug interruptions in this group of patients as well as to assess its safety and potential changes in health-related quality of life (HR-QOL).

In summary, TB-IRIS is a common complication of ART in patients with HIV-associated TB. The risk of TB-IRIS is highest in patients with the lowest CD4 counts; rapid ART initiation in such patients (essential because of the proven survival benefit) increases this risk even further. Immunological studies have shown elevation of a range of proinflammatory cytokines at the time of TB-IRIS onset [22,24-26]. Corticosteroids have proven to be beneficial in the treatment of TB-IRIS. This effect is likely due at least partially to reductions in blood concentrations of key cytokines mediating the condition. We are therefore evaluating whether prednisone could also be beneficial in preventing TB-IRIS. Because in the majority of cases the onset of TB-IRIS is within the first 4 weeks of ART [27], prophylactic interventions may only be required for one month. Adjunctive corticosteroids in these profoundly immunosuppressed patients might not be without risk; they have been shown to increase the risk of Kaposi sarcoma and herpes reactivations [15,18-20], mainly in patients not on ART. All patients in our trial are receiving ART, which is likely to be at least partially protective against these adverse effects.

## Methods

### Study Design

This is a proof-of-concept, phase III, randomized, double-blind, placebo-controlled trial of prednisone to assess its efficacy and safety in preventing paradoxical TB-IRIS in high-risk patients starting ART. The intervention is oral prednisone 40 mg daily for 14 days started within 48 hours of initiating ART, followed by 20 mg daily for 14 days. Based on the average weight of participants, this is a lower dose than was used in our previous TB-IRIS prednisone treatment trial [16]. Our reasoning was that when using prednisone as prophylaxis (rather than as antiinflammatory treatment), a lower dose would be potentially effective with a lower risk for adverse events. In our decision regarding the prednisone dose for this trial, we factored in that rifampicin increases the clearance of prednisolone by 45% [28].

### Sample Size Calculation

The incidence of paradoxical TB-IRIS in patients on TB treatment starting ART varies between 0% and 54% in the literature. In a recent meta-analysis, the pooled estimate was 18% (95% CI 16%-21%) [2]. However, the incidence is substantially higher in patients with a CD4 count ≤100/μL starting ART within 30 days of TB treatment because these are two major risk factors [2,29]. Assuming 35% cumulative incidence of TB-IRIS in the placebo arm and a 50% relative reduction in the prednisone arm and requiring 80% power to test for the difference in TB-IRIS at a 2-sided significance level of 5%, the sample size required was 110 in each arm. We therefore aimed to recruit 240 participants, assuming loss to follow-up of 10%.

### Allocation

Blinded medication containers were packaged at an independent off-site pharmacy in a 1:1 randomization sequence with block sizes of 8. The sequence was prepared before the trial started by an independent statistician. The packages contain either prednisone 5 mg or identical placebo tablets. Each package has a number from 1 to 240. Participants are enrolled sequentially and receive the next study number from 1 to 240 with the corresponding medication package. Participants and all study staff remain blinded to the treatment allocation throughout the course of the trial. When deemed essential for ongoing clinical management by the attending clinician, unblinding of the randomization allocation can occur.

### Setting, Selection, and Enrollment of Participants

**Figure 1:**
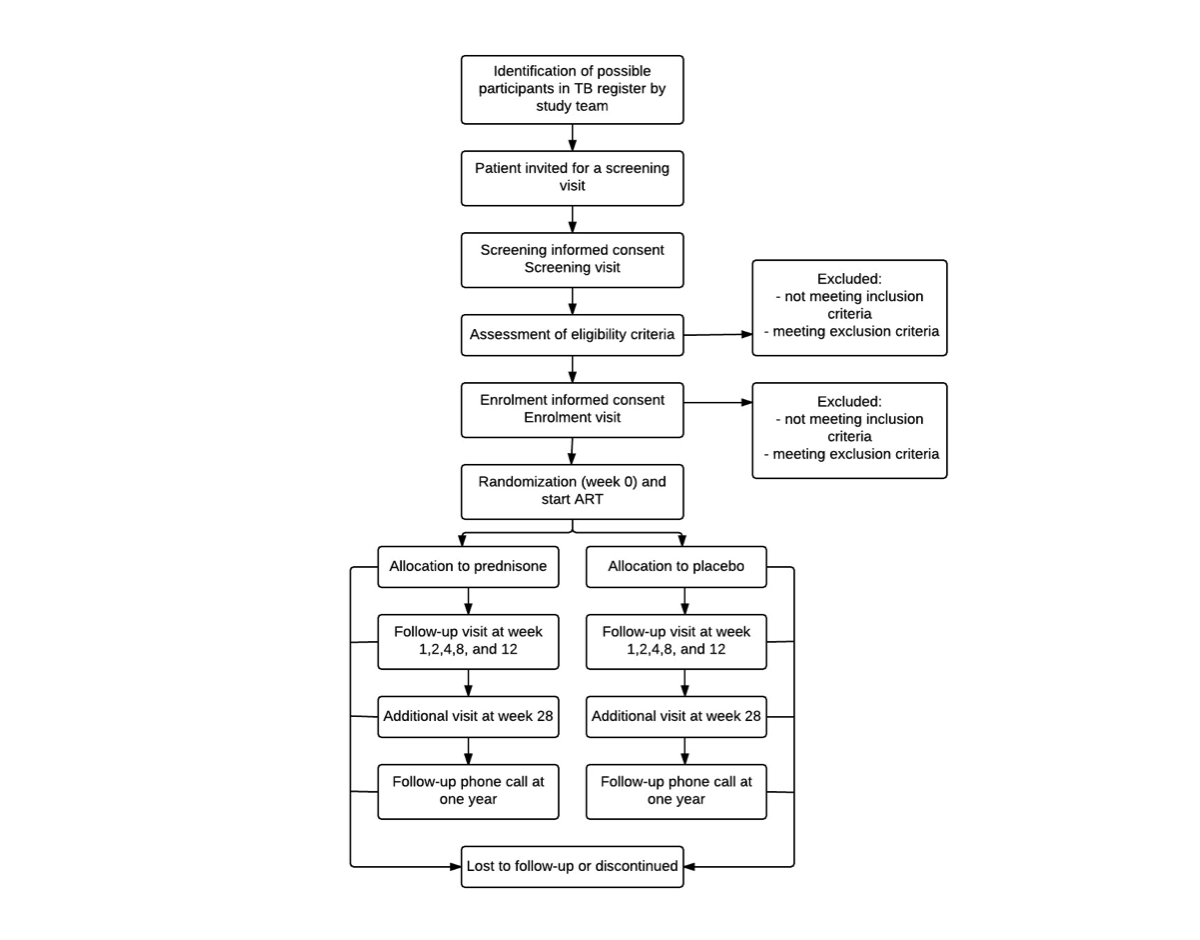
Flow of participants.

Participants are recruited from four different TB clinics in Khayelitsha, a township 20 kilometers from Cape Town’s center with an estimated 500,000 inhabitants. An estimated 16% of the population is HIV infected [30]; TB case notification is 917/100,000 per year and HIV coinfection among TB cases is 60% (City of Cape Town, 2015). The study team identifies patients who could fulfill the enrollment criteria and invites them to attend a screening visit. Informed consent is obtained for the screening visit in the language of choice (mainly isiXhosa), an information leaflet about the trial is provided and discussed, and a screening visit is conducted. Patients who fulfill enrollment criteria are invited to enroll in the trial. Detailed information about the trial is provided by a member of the study team, who also answers all questions the patient has before enrollment. An enrollment informed consent form is signed if the patient agrees to participate in the trial. In patients who are unable to write, informed consent is taken in the presence of an independent witness, who signs the informed consent document next to the participant’s thumb print. Participation is voluntary and patients can refuse participation or withdraw from the trial at any time without compromising their standard medical care (See Figure 1).

### Inclusion and Exclusion Criteria

Inclusion and exclusion criteria are shown in Textbox 1. Exclusion criteria include conditions where corticosteroid treatment is recommended and conditions in which corticosteroids are contraindicated or potentially harmful. Patients should be receiving standard intensive-phase treatment for drug-susceptible TB (rifampicin [RIF], isoniazid [INH], pyrazinamide [PZA], and ethambutol [EMB]) when enrolled in the trial.

**Inclusion and exclusion criteria.**

Inclusion criteria:

- Age 18 years or older
- HIV-infected
- CD4 count ≤100/μL (in the past 3 months)
- ART-naïve
- Confirmed diagnosis of TB or strong clinical and radiological evidence of TB with symptomatic response to TB treatment
- On TB treatment for less than 30 days prior to study entry
- Eligible for ART and patient consents to start ART within 30 days of starting TB treatment
- Written informed consent

Exclusion criteria:

- Kaposi sarcoma
- Pregnant
- TB meningitis or tuberculoma at TB diagnosis
- Clinical syndrome of pericardial TB at TB diagnosis
- Rifampicin-resistant TB
- On corticosteroids for another indication or on any other immunosuppressive medication within the past 7 days
- Uncontrolled diabetes mellitus
- Alanine aminotransferase >200 IU/L
- Absolute neutrophil count <500/μL
- Not on standard intensive-phase TB treatment
- Poor clinical response to TB treatment prior to ART as judged by the clinical investigators
- Hepatitis B surface antigen positive

### Study Endpoints

The primary endpoint is the development of paradoxical TB-IRIS within 12 weeks of starting ART, defined using the International Network for the Study of HIV-associated IRIS (INSHI) consensus case definition [31]. Secondary endpoints are divided into efficacy and safety and tolerability endpoints (Textbox 2).

**Secondary endpoints.**

Secondary efficacy endpoints:

- Time to TB-IRIS event from start of ART
- Severity of TB-IRIS events (defined by need for hospitalization, neurological involvement, and C-reactive protein)
- Duration of TB-IRIS events
- Mortality attributed to TB and TB-IRIS
- All-cause mortality
- Composite endpoint of death, hospitalization, or hepatotoxicity
- Other (non-TB) IRIS events
- Health-related quality of life assessments using the Patient-Reported Outcomes Quality of Life–HIV (PROQOL-HIV) [32], an adaptation of the HIV symptom index [33], and the EuroQol Five Dimensions Questionnaire (EQ-5D-3L) [34]
- Adverse events and severe adverse events ascribed to TB treatment, ART, or co-trimoxazole
- Discontinuation of ART or TB treatment for more than 5 days due to adverse events
- Number of hospitalizations and total days hospitalized

Secondary safety and tolerability endpoints:

- Corticosteroid-associated adverse events (predefined as hypertension, hyperglycemia, hypomania/mania, depression, acne, epigastric pain, upper gastrointestinal bleeding, Cushingoid features, new edema, and avascular bone necrosis)
- Laboratory safety data: glucose, full blood count, and electrolytes
- Other infections and malignancies
- All grade 1, 2, 3, and 4 adverse events using the Division Of AIDS Table For Grading the Severity of Adult and Pediatric Adverse Events [35]

### Medication

Study medication is prednisone tablets (5 mg) or identical placebo. The study medication was manufactured by the Gulf Drug Company in Durban, South Africa; this company supplies prednisone (Trolic) to the South African government hospital pharmacies, and the product is registered with the Medicines Control Council of South Africa. Participants receive 8 tablets daily (40 mg prednisone or placebo) for 14 days followed by 4 tablets daily (20 mg prednisone or placebo) for 14 days. Study medication is dispensed at week 0 (for the first 14 days) and at week 2 (for the next 14 days). During these first four weeks of the trial, concomitant treatment with nonsteroidal anti inflammatory drugs, any systemic corticosteroid medication, or any other immunosuppressive medication or chemotherapy is prohibited. The protocol requires that the study drug be stopped in the following situations: Kaposi sarcoma or new World Health Organization stage 4 opportunistic condition diagnosed, diagnosis of rifampicin-resistant TB, development of TB-IRIS requiring open label prednisone, requirement for prohibited concomitant medication, diagnosis of pregnancy, request by the participant, clinical reasons believed to be life threatening by the trial doctor, or interruption of ART or study drug for more than five days by the participant or by clinician (eg, in the event of ART toxicity).

ART is provided according to the South African Department of Health guidelines [36]. First-line ART consists of tenofovir (TDF) 300 mg daily, emtricitabine (FTC) 200 mg daily, and efavirenz (EFV) 600 mg daily. In case of contraindications or toxicity, TDF is substituted with abacavir (ABC) and EFV with nevirapine or lopinavir boosted with ritonavir (LPV/r). LPV/r is double-dosed in patients taking rifampicin [37]. ABC only became available for use in the public sector during the course of our trial. Before its availability, TDF was replaced with either zidovudine or stavudine in the event of contraindication or toxicity. The intensive follow-up period of the trial is 12 weeks; no switches to ART for virological failure are made during this period. Participants receive ART from their ART clinic in close communication with the study team.

TB treatment is prescribed according to South African Department of Health guidelines [38] by the participant’s TB clinic. Participants receive weight-based daily doses of INH, RIF, PZA, and EMB for 2 months, followed by INH and RIF for another 4 months. If TB drug-induced liver injury occurs during follow-up, participants are managed according to local clinical guidelines. In short, TB treatment is stopped and replaced with 3 drugs with no/low hepatotoxicity risk (eg, EMB, kanamycin, and moxifloxacin). In addition, other possible hepatotoxic drugs like co-trimoxazole and EFV may be temporarily stopped or replaced. Once symptoms of hepatitis have resolved and alanine aminotransferase is <100 IU/L, drugs can be rechallenged one by one with close monitoring of liver enzymes. Duration of TB treatment is individualized after rechallenge. All participants are eligible for co-trimoxazole prophylaxis unless contraindicated.

### Schedule

Study visits occur at screening, enrollment, week 0 (the day the participant starts study drug and ART), week 1, week 2, week 4, week 8, and week 12. A window period of 4 days is allowed for each visit from week 1 to week 8; week 12 has a 7-day window. Assessments done at each visit are summarized in Table 1. If the attending clinician suspects TB-IRIS, laboratory investigations including a bacterial blood culture and a chest radiograph are performed.

Outside the scheduled visits, participants can attend for unscheduled visits if they experience symptomatic deterioration or if deemed necessary by the attending clinician. For participants with ongoing TB-IRIS at week 12, follow-up is extended in order to ascertain the end date of TB-IRIS.

**Table 1:** Schedule of events.

Study visit	Screening	Enrollment	Wk 0	Wk 1	Wk 2	Wk 4	Wk 8	Wk 12	Unscheduled visit
ART^a^ day	Not specified	Aim for −7 to 0	0	7±4	14±4	28±4	56±4	84±7	Not specified
Document HIV status	x								
Screening ICF^b^	x								
Enrollment ICF		x							
Study drug dispensed			x		x				
Symptoms^c^	x	x	x	x	x	x	x	x	x
Karnofsky score	x		x	x	x	x	x	x	x
Pill count^d^				x	x	x	x	x	x
HR-QOL^e^ assessments			x			x		x	
Examination	x	I^f^	x	x	x	x	x	x	x
Laboratory investigations^g^	x	I	x		x	x		x	I
CD4 count, HIV viral load	x							x	
Serum HBsAg^h^	x								
Serum CrAg^i^	x								
Urinary pregnancy test	x	I	I	I	I	I	I	I	I
Storage bloods and immunology assays			x		x	x		x	If IRIS suspected
Storage urine			x					x	If IRIS suspected
Chest radiograph	I		x						If IRIS suspected
Sputum Xpert MTB/RIF^j^, TB culture, and DST^k^	x					x		x	
Initiate ART			x						

^a^ART: antiretroviral therapy.

^b^ICF: informed consent form.

^c^Symptoms and specific screening for adverse events and TB-IRIS.

^d^Pill count: ART and study drug week 1-4.

^e^HR-QOL: health-related quality of life.

^f^I: if clinically indicated.

^g^Laboratory investigations: full blood count with leucocyte differentiation, sodium, potassium, creatinine, glucose, bilirubin, alanine aminotransferase, alkaline phosphatase, C-reactive protein.

^h^HBsAg: hepatitis B surface antigen.

^i^CrAg: cryptococcal antigen.

^j^MTB/RIF: Mycobacterium tuberculosis, resistance to rifampicin.

^k^DST: drug sensitivity testing.

### Protocol Change With Additional Safety Assessments

In the initial protocol, participant follow-up ended at week 12 unless there was ongoing TB-IRIS at this visit. A year after the start of our trial, the results of the IMPI trial [15] became available, showing an increased incidence of cancer in patients with HIV-related TB pericarditis prescribed prednisolone compared to those prescribed placebo. This was largely attributable to an increase in HIV-associated cancers (Kaposi sarcoma and non-Hodgkin lymphoma). The potential risk of Kaposi sarcoma associated with corticosteroid use in HIV-infected patients was known and addressed in our protocol prior to the IMPI publication: Kaposi sarcoma at screening is an exclusion criterion, Kaposi sarcoma is ascertained as a safety endpoint, and if Kaposi sarcoma occurs in patients on the trial this is an indication to immediately stop study medication.

As a study team we reviewed the implications of the IMPI findings for safety of participants in our trial. A number of considerations make the IMPI trial different from the PredART trial with respect to the risk of Kaposi sarcoma. The prednisone dose used in PredART is substantially lower and of shorter duration. All participants in the PredART trial are started on ART when they start study medication, and ART is known to reduce the risk of Kaposi sarcoma [39]; in contrast, in IMPI, 7 out of 9 of the prednisolone-treated patients who developed HIV-related cancers were not on ART at the time of enrollment and commencement of steroids (personal communication, IMPI investigators). Lastly, the PredART trial participants are not being randomized to *M indicus pranii*; this is relevant, as most of the cancers were diagnosed in participants receiving both prednisolone and *M indicus pranii* in the IMPI trial.

At the time of the IMPI publication, 89 participants had been randomized in our trial and none had developed Kaposi sarcoma. We communicated these findings and considerations to the PredART trial data safety and monitoring board (DSMB), which advised that the PredART trial should continue but that we should add extra visits for safety assesments. We added one visit at week 28 and a telephonic follow-up at one year to ascertain HIV-related cancers. During the week 28 visit, patient history is taken and clinical examination is performed specifically to assess for any history, symptoms, or signs of cancer. One year after starting ART, participants are phoned by the study team and their clinical records are assessed to review if there has been a new diagnosis of cancer subsequent to the week 28 visit. The protocol was amended accordingly and the information given during the informed consent process was updated with approval by the relevant ethics committees. The additional assessments applied to all participants, including those who had already completed their 12-week follow-up at the time of this protocol change.

### Management of Tuberculosis-Associated Immune Reconstitution Inflammatory Syndrome

TB-IRIS is a diagnosis of exclusion. When TB-IRIS is suspected, investigations are performed with the main aim to exclude alternative causes of clinical deterioration. For all participants with suspected TB-IRIS, these include the laboratory investigations listed in Table 1, a bacterial blood culture, and a chest radiograph. Other investigations (eg, abdominal ultrasound, lumbar puncture) are undertaken when appropriate based on clinical presentation. Adherence to ART and TB treatment is assessed. If TB-IRIS is diagnosed (fulfilling the INSHI criteria [31]), the study drug is stopped, and open label prednisone is started at a dose of 1.5 mg/kg/day and weaned over 4 weeks or longer depending on clinical response.

### Other Aspects of Clinical Management

Mild drug rashes are closely monitored with symptomatic therapy. If more severe drug rashes occur, potential causative drugs (TB medication, EFV, co-trimoxazole) are stopped and rechallenge occurs following local guidelines. All other clinical management, including treatment of new opportunistic infections, malignancies, and comorbidities, is according to South African National Department of Health guidelines.

### Health-Related Quality of Life Assessments

Health-related quality of life (HR-QOL) is measured using three different questionnaires: the Patient-Reported Outcomes Quality of Life–HIV (PROQOL-HIV) [32], an adaptation of the HIV symptom index by Justice [33], and the more generic EuroQol Five Dimensions Questionnaire (EQ-5D-3L) [34]. Participants complete all three questionnaires at week 0, week 4, and week 12. Our aim is to assess HR-QOL in patients with HIV-associated TB who have recently started treatment for both conditions and to find which questionnaire best captures their HR-QOL in the acute phase of their illness. Since the PROQOL-HIV has not been used in populations with TB-IRIS in comparable settings, these data will serve to validate this instrument through triangulating the results of debriefing interviews and the two other HR-QOL instruments. Moreover, we are assessing the impact of TB-IRIS and prednisone use on HR-QOL.

### Tuberculosis-Associated Immune Reconstitution Inflammatory Syndrome Endpoint Evaluation

An endpoint review committee has been established to review all cases of suspected TB-IRIS and determine whether they fulfill the INSHI case definition. This committee is comprised of 3 expert clinical investigators not active at the clinical site. Reviewers will have access to all clinical and laboratory data entered in the database, including chest radiographs and TB-IRIS narrative summaries written by the attending clinician, and they will be blinded to treatment allocation. The review will take place after completion of the clinical trial. Each reviewer will independently review the summaries and data submitted to him or her, using the INSHI paradoxical TB-IRIS case definition [31]. For those cases where there is disagreement, consensus will be sought at a meeting where investigators will also be present to provide additional clinical information when needed. If consensus cannot be reached a 2-1 vote will decide.

### Statistical Analysis

The statistical analysis of the trial will be performed according to a plan prespecified before the database is locked and allocation unblinded. The primary analysis and secondary efficacy analysis will be performed using an intention-to-treat approach; safety analysis will be performed using the all-patients-treated approach. The primary endpoint will be tested comparing the proportion of patients diagnosed with paradoxical TB-IRIS among treatment groups using a Fisher exact test. Secondary endpoints will be analyzed comparing study arms with the Fisher exact test (for categorical data) or Wilcoxon rank-sum test (for continuous data). Time to event analysis will be used to compare time from the start of ART to TB-IRIS between study arms. A prespecified subgroup analysis of those patients with a baseline CD4 count <50/μL will be performed. Adverse events will be coded following the Medical Dictionary for Regulatory Activities (MedDRA). Safety endpoints will be compared between both arms individually (eg, analysis comparing development of hyperglycemia in each arm) as well as collectively (eg, analysis comparing all corticosteroid side effects that occurred in each arm).

### Data Handling and Record Keeping

The identity and information of trial participants is kept confidential. All relevant clinical information to reconstruct and evaluate the trial is kept as source documents. Data from source documents are entered into a Web-based electronic database specifically developed for the trial. The database is access-controlled and data deidentified. Point-of-entry data validation and a double–data entry system with discrepancy reporting are key measures to ensure data integrity. After completion of the first 12 weeks of the study, the electronic record of each participant is reviewed against the case report form. After completion hereof, aggregate database entries are checked for outlying data, missing data, and systematic errors according to the prespecified data management plan.

### Monitoring, Oversight, and Reporting

The trial is sponsored by the University of Cape Town. which has subcontracted an independent clinical trial monitor who conducts monitoring visits once every 1 to 2 months and reports to the sponsor. An independent DSMB reviewed study recruitment, data quality, and safety endpoints twice during the trial—after 80 participants had completed their week 12 visit and again after 160 participants had completed their week 12 visit. The DSMB was constituted and conducted its tasks according to the study-specific DSMB charter. It was required to advise the sponsor of any major safety and data quality issues. At both interim reviews, the DSMB advised that trial recruitment and follow-up should continue. All adverse study drug reactions, serious adverse events, and deaths are reported to the sponsor, the University of Cape Town Human Research Ethics Committee, the Institute for Tropical Medicine Institutional Review Board, and the Medicines Control Council according to their respective reporting guidelines.

### Ethical Approval

The protocol was approved by the University of Cape Town Human Research Ethics Committee (HREC 136/2013), Institute of Tropical Medicine Institutional Review Board (882/13), and the Antwerp University Hospital Ethical Committee (13/20/224). The same approval schedule is applicable to protocol amendments. The trial is conducted according to International Council for Harmonisation Good Clinical Practice standards [40], the South African Good Clinical Practice guidelines [41], and the Declaration of Helsinki [42].

### Funding

This trial is funded by the European and Developing Countries Clinical Trials Partnership through a Strategic Primer Grant (SP.2011.41304.074) that was awarded to the University of Cape Town, the Institute of Tropical Medicine, and Imperial College London. There is cofunding from the South African Government Department of Science and Technology, the Wellcome Trust (098316, 084323, 104803), the Medical Research Council of South Africa, and a PhD fellowship from the Institute for Tropical Medicine.

## Results

The trial started enrollment at the end of August 2013; 240 participants are currently enrolled according to plan. Follow-up for the first 12 weeks was completed in June 2016, and the final safety assessments for the trial will be completed by March 2017.

## Discussion

With guidelines advising that ART be started with minimal delay during treatment for HIV-associated TB, and many patients still presenting with a low CD4 count, TB-IRIS will remain a major complicating factor in patients with HIV-associated TB starting ART in sub-Saharan Africa. If corticosteroids are shown to reduce the risk of TB-IRIS without safety concerns, clinicians will have an evidence-based preventive option in patients at high risk for TB-IRIS provided they do not have a contraindication for corticosteroids. Therefore, we anticipate that the findings of this clinical trial could inform clinical practice and guidelines in sub-Saharan Africa and internationally.

## Abbreviations

ABC: abacavir
ART: antiretroviral therapy
CrAg: cryptococcal antigen
CRP: C-reactive protein
DSMB: data safety and monitoring board
DST: drug sensitivity testing
EFV: efavirenz
EMB: ethambutol
EQ-5D-3L: EuroQol Five Dimensions Questionnaire
FTC: emtricitabine
HBsAg: hepatitis B surface antigen
HR-QOL: health-related quality of life
ICF: informed consent form
IMPI: Investigation of the Management of Pericarditis
INH: isoniazid
INSHI: International Network for the Study of HIV-associated IRIS
LPV/r: lopinavir boosted with ritonavir
PROQOL-HIV: Patient-Reported Outcomes Quality of Life–HIV
PZA: pyrazinamide
RIF: rifampicin
TB: tuberculosis
TB-IRIS: tuberculosis-associated immune reconstitution inflammatory syndrome
TDF: tenofovir

## Appendix

Multimedia Appendix 1 Review by funders (EDCTP).

Multimedia Appendix 2 Response to EDCTP.
