# Supplementary material for: Preventing Paradoxical Tuberculosis-Associated Immune Reconstitution Inflammatory Syndrome in High-Risk Patients: Protocol of a Randomized Placebo-Controlled Trial of Prednisone (PredART Trial)
Source: JMIR Res Protoc. 2016 Aug 29;5(3):e173. doi: 10.2196/resprot.6046 (PMC5020311; doi:10.2196/resprot.6046)
Supplement: Multimedia Appendix 2 [file resprot_v5i3e173_app2.pdf]

IIDMM
INSTITUTE OF INFECTIOUS DISEASE AND MOLECULAR MEDICINE

Graeme Meintjes MBChB MRCP(UK) FCP(SA) DipHIVMan(SA) PhD
Associate Professor
Institute of Infectious Diseases and Molecular Medicine, Faculty of Health Sciences,
University of Cape Town, Anzio Road, Observatory 7925, South Africa
email: graemein@mweb.co.za Phone: +27 (0) 21 4066797

10 July 2012

Wendy Morrill
Administrative Officer
European and Developing Countries Clinical Trials Partnership (EDCTP)
P.O. Box 93015
2509 AA
The Hague
The Netherlands

Dear Ms Morrill

RE: SP.2011.41304.074 - ***Preventing tuberculosis-associated immune reconstitution inflammatory syndrome in high-risk patients: a randomised placebo-controlled trial of prednisone:*** **RESPONSE TO REVIEWERS**

Please find our responses to the reviewers on the 2 pages that follow. We would like to thank the four reviewers for their constructive comments and we are grateful for the opportunity to respond. We firstly address two general points, then address specific questions and major points raised by the reviewers.

Thank you for considering our application further.

Yours sincerely

Graeme Meintjes
MBChB MRCP(UK) FCP(SA) DipHIVMan(SA) PhD
Infectious Diseases Physician

"Our Mission is to be an outstanding teaching and research university, educating for life and addressing the challenges facing our society."

General issues:

1) Research to prevent TB-IRIS is a priority and we think it fits well with the mission, aims and objectives of EDCTP. Treating HIV and TB simultaneously in the same patient is a major clinical challenge for clinicians in Africa. Paradoxical TB-IRIS is the most frequent complication and results in substantial numbers of hospital admissions, consumes health care resources, the mortality associated with TB-IRIS in routine clinical practice is not established, it is predicted that TB-IRIS will become more common with guidelines shifting to earlier ART initiation and there is at present no evidence-base for TB-IRIS prevention. We propose the use of an established drug (prednisone) as a new strategy (prevention of TB-IRIS) in patients with TB and HIV, two infectious diseases that are highlighted by the EDCTP as priority research areas. Furthermore we highlight capacity development and networking as key aspects of this project.

2) Simplicity in design and conduct of this proof-of-concept clinical trial was prioritized. We are mindful that a maximum of 2 years funding is available and have thus designed the study with only 2 arms (prednisone and placebo) and endpoints that can be readily ascertained. Complicating the design of this study with multiple arms/doses and introducing pharmacological/immunological endpoints will undermine this and make it very difficult to obtain answers to the research questions within the 2-year timeframe. We do propose storage of samples for later immunology studies (discussed below).

Reviewer 1:

"*The investigators describe this as a Phase II trial designed primarily for safety and yet the primary endpoint is one of efficacy not safety*." Response: The study is clearly labelled as a proof-of-concept RCT designed to address both efficacy and safety. The primary efficacy endpoint is the development of paradoxical TB-IRIS, and safety is ascertained through adverse events and laboratory safety measures that are secondary endpoints. We do not see the study as primarily a safety study nor do we describe it as such.

"*Given the superb track record of the investigator team in pharmacology and immunology and that they plan a larger trial with a mortality end point (a great idea) why not use this study to study a) series of different doses (and or durations) of the steroids b) detailed analysis of the effects and analysis of the impact of different doses on PK/PD and dissection of the immune responses at different doses and use that data to design the ideal large pragmatic Phase III trial with a mortality end point*." Response: Firstly, please see our point about simplicity above. Secondly, our approach to the important issues raised by the reviewer is to address these issues in a different order. We plan first to demonstrate proof-of-concept that steroids are of clinical benefit in TB-IRIS. We have chosen a dosing schedule that represents a mid-range for anti-inflammatory effect. If clinical benefit is shown we will then proceed to address immunological questions. We will obtain separate funding for such a sub-study. Indeed we plan to store specimens for immunology studies (plasma for Luminex; PBMC for FACS) that will allow us to study mechanisms of action. This is what we did in our RCT of TB-IRIS treatment and results of the immunology sub-study have been published recently (Meintjes et al, AJRCCM, Epub ahead of print). Including dose ranging in the design would complicate the study and increase the required sample size and cost and immunological and radiographic endpoints may not be sufficiently discriminatory to differentiate between doses. We do not feel this is the correct trial to study the PK/PD of steroids in TB: researching correlates between drug concentration and effect would best be done in a treatment rather than prevention study. Furthermore, we think that the alternative endpoints proposed are soft (immunology, imaging, etc.) and would not be sufficient evidence to impact clinical practice.

"*The sample size calculation looks underdone to me! They have chosen an incidence of IRIS at the upper end of the spectrum and are hoping for a huge effect of steroids. I worry they will get a grey result and run the risk of missing an effect and putting at risk the plans for the larger trial*." Response: We do not agree that we overestimated the TB-IRIS incidence given the patient population we are targeting. The estimate of 35% in the placebo arm corresponds to the findings of Blanc et al (110/332 (33%) of participants who started ART in the early arm had TB-IRIS events (Blanc, NEJM 2011)) and a study we conducted at Brooklyn Chest Hospital in Cape Town (42% IRIS incidence, Van der Plas, submitted). Our estimate reflects

that patients will be eligible for our RCT precisely because they are at high risk for TB-IRIS (CD4 count ≤100 and short interval between TB treatment and ART start (<30 days)). It is true that the estimated 50% effect size for steroids is high, but given the effect of steroids in the treatment of IRIS and that in a randomized trial in TB meningitis, 8 cases of severe hepatitis (one fatal) occurred in the placebo group and none in the dexamethasone group we anticipate a substantial effect of steroids on inflammatory symptoms and quality of life (probably not on mortality but this is not the endpoint in the initial study). We appreciate the concern of the referee that a generally supportive but statistically non-significant ("grey") result would jeopardize the plans for a larger trial. We will revise our plans during conduct of the initial study by using adaptive triangular design, defining regions where we would stop for futility (insufficient preventive effects) and superiority (significant preventive effect). For example, if the preventive effect is shown to be <20% incidence reduction, we may stop the study for futility. If the effect, at an interim analysis, shows a statistically significant effect, we will proceed to seek funding for the larger phase 3 trial immediately. Full details will be described the study protocol. We will consider a seamless phase 2/3 design, allowing the use of the phase 2 patients in the phase 3 mortality study. However, given that the maximal sample size will need to be decided in advance, the triangular design may still result in an undetermined result. This will occur if the observed effect is <35% with a TB-IRIS incidence in the upper range amongst controls (30 to 50%) or <50% with a TB-IRIS incidence in the lower range amongst controls (20 to 30%). Progress to a phase 3 study will then be determined on the basis of effect size and incidence estimates. The secondary and safety endpoints will also inform this decision based on risk/benefit analysis.
The reviewer suggests we may be guilty of selective reporting of the literature. We have excluded the 2 publications mentioned because: the Vietnam trial included only TB meningitis patients (TBM is an exclusion criteria in our trial) and the Zimbabwe trial was restricted to patients with cryptococcal meningitis. Regarding the DSMB, we will approach experts in the field and request them to be members of the DSMB immediately if funding is granted. We intend to ask: Frank Cobelens, Guy Thwaites and Gavin Churchyard in addition to the statistician. We will also be also be approaching Bill Burman (University of Colorado and head of US CDC/IDSA HIV-TB guidelines writing committee) to ask if he would be prepared to sit on the Trial Steering Committee. He has vaste clinical and trials experience in HIV-TB.

Reviewer 2:
"The proportion of time committed by some co-investigators is rather limited" Response: It is true that for certain co-investigators this is limited, but this is a realistic commitment from senior academics who have many other commitments and whose main role on this trial is to serve on the committees described and mentor the PI, Graeme Meintjes, who has committed 60% time and will manage and oversee the trial site research team with advice/support from the ITM Clinical Trials Unit (who have substantial experience with EDCTP trials).
"The proposal might not completely fit the mission and objectives of EDCTP." Response: We do not understand the reason for this comment. Our trial aims at improving the care and treatment of HIV and TB, infections that very frequently occur together in Africa. Please also see general comment above.

Reviewer 3:
"Fits very well, only issue is that this is a treatment trial for the management of TB-IRIS and so may not be core to the aims of EDCTP". TB-IRIS has been recognized by EDCTP as an important research area since 2005. In 2006 an international IRIS workshop was sponsored by EDCTP in Kampala. This workshop was very successful and lead to the establishment of the International Network for the Study of HIV-associated IRIS (INSHI). This network has developed several case definitions for IRIS and organized many additional scientific meetings. It was realized from the start that to perform trials on HIV and/or TB in countries where co-infection is highly prevalent we need to have evidenced-based strategies to diagnose, predict, prevent and treat IRIS.

Reviewer 4:
There do not appear to be questions/issues raised for us to address.
