# Supplementary material for: Preventing Paradoxical Tuberculosis-Associated Immune Reconstitution Inflammatory Syndrome in High-Risk Patients: Protocol of a Randomized Placebo-Controlled Trial of Prednisone (PredART Trial)
Source: JMIR Res Protoc. 2016 Aug 29;5(3):e173. doi: 10.2196/resprot.6046 (PMC5020311; doi:10.2196/resprot.6046)
Supplement: Multimedia Appendix 1 [file resprot_v5i3e173_app1.pdf]

E D C T P

Dr Graeme Meintjes
University of Cape Town
Wolfson Pavilion Level 3, IIDMM, UCT Faculty of Health Sciences
7925 Observatory
South Africa

Date: 6 July 2012
Phone: +31(0)70 344 0880
Fax: +31(0)70 344 0899
E-mail: proposals@edctp.org
Web: www.edctp.org

Ref. Nr. 64193

Subject: **Feedback from peer-review of full application SP.2011.41304.074** - ***Preventing tuberculosis-associated immune reconstitution inflammatory syndrome in high-risk patients: a randomised placebo-controlled trial of prednisone***

Dear Dr Meintjes,

Thank you for submitting your application to the EDCTP Strategic Primer Grant Call for Proposals. Your application has been assessed by external reviewers and I enclose their comments for your attention. Please respond to any major issues and questions raised, limiting your response to two A4 pages (font Verdana size 10 or equivalent font/size). Please note that we expect to receive an additional review and I will send this to you if and when it is submitted.

In order to ensure that the Scientific Review Committee has the opportunity to consider your response before its meeting on 16 July, please submit your response as an attachment (pdf format on institutional letterhead paper) by email to proposals@edctp.org with the following name format in the e-mail subject line: *Applicant's Surname – Project Code – Feedback Response* by **10 July 2012**.

Thank you in advance for your cooperation and I look forward to receiving your response in due course.

Sincerely,

Jean Marie Vianney Habarugira
Project Officer, EDCTP

Enclosed: Peer-review feedback

Reviewer 1:

- A superb team ideally set up to undertake the study. Clear commitment from the applicants to the study. Good balance of European and African partners and appropriate gender balance. No concerns about the ability of the applicants to deliver – they are world leaders in this field.
- Very important study which addresses a key issue in global health. The key questions are clearly articulated. The proposal will address the questions posed. Yes the proposal will deliver important insights.
- I would like to suggest to the applicants to consider adapting the design to yield greater information.

  1: The investigators describe this as a Phase II trial designed primarily for safety and yet the primary endpoint is one of efficacy not safety. The design seems somewhat schizophrenic caught between a safety study and an efficacy study and as such may fail to deliver either, too small to convince on the effects, a somewhat missed opportunity to sort out the pharmacology and correlates of effect – particularly given the track record of the applicants.

  2: Given the superb track record of the investigator team in pharmacology and immunology and that they plan a larger trial with a mortality end point (a great idea) why not use this study to study a) series of different doses (and or durations) of the steroids b) detailed analysis of the effects and analysis of the impact of different doses on PK/PD and dissection of the immune responses at different doses and use that data to design the ideal large pragmatic Phase III trial with a mortality end point.

  3: The sample size calculation looks underdone to me! They have chosen an incidence of IRIS at the upper end of the spectrum and are hoping for a huge effect of steroids. I worry they will get a grey result and run the risk of missing an effect and putting at risk the plans for the larger trial.
  Why not back step, assess a series of doses (and possibly durations), interrogate the biological correlates of the steroids (standard cytokine/chemokine assays, arrays, expression, and imaging) and rationally choose the optimal dose for the large trial, which is clearly needed? In the unit where the team is based they have access to some of the best clinicians, pharmacologists and immunologists working in TB globally a little disappointing not to see them more integrated with the clinical study. I think they could be more ambitious in what they get out of this Phase II study.

- Not only would it be great to see more pharmacology and immunology but perhaps also an opportunity to interrogate the recent work on the effects of steroids in TB - Cell. 2012 Feb 3; 148(3):434-46.

- I am very supportive of the work but I just wonder if there is a chance for the team to consider a slightly different design in what is a Phase II study and gain greater insight to plan the larger definitive trial powered with a mortality endpoint.

- The investigators a little guilty of selective reporting of the literature on the timing of ARVs, outcomes and IRIS – references 31-33 perhaps should be balanced by the studies from Vietnam (Clin Infect Dis. 2011 Jun; 52(11):1374-83.) and Zimbabwe (Clin Infect Dis. 2010 Jun 1;50(11):1532-8) on timing of ARVs and outcome in opportunistic infections in HIV co-infected individuals.

- Would be good to see who was on the DSMB. I am not used to the TSC being made up of investigators so directly linked with the study and wonder if there would be scope for independent members and/or chair.
- I think this application from one of the world's leading TB Clinical Research Groups is excellent. I have made some suggestions for the applicants to consider which I hope are helpful. But the comments are for consideration. My personal view is that they may help improve the study and future studies.

Reviewer 2:

- The research group has the quality and competencies needed to undertake the proposed project to accomplish their targets given their documented experience and expertise. Investigators have expertise across disciplines, but the proportion of time committed by some co-investigators is rather limited – could this perhaps compromise quality? The team balance in terms of European and African partners is reasonable, but not quite with regard to gender.
- The research questions and hypotheses have merit. The rationale of the project is based on an important health issue given that paradoxical TB-IRIS occurs in 8 to 43% of patients (HIV-infected patients) starting antiretroviral therapy (ART) while receiving TB treatment. The proposal addresses an issue of considerable importance to human health and the anticipated findings could translate into clinical practice.
- The objectives and study design are clear, of a high standard, and appropriate to the research questions. The experimental plans are realistic, given the aims of the research and the resources. No unusual complications or limitations are anticipated considering that the researchers have extended experience in the subject area. The proposal has a high probability of successful accomplishment. The suggested duration of the project is rather ambitious, but could be achieved; estimated time frame of clinical trial: 1 March 2013 to 30 June 2014.
- The aim of this project is to use the results to inform design and sample size calculation for a larger phase 3 trial. The proposal will contribute new knowledge in the subject area and the results should have important clinical implications and the potential to influence policy.
- The proposal will not accelerate the development of new drugs, but may improve treatment strategies. The knowledge gained will contribute to a better understanding of the interaction between HIV and TB medications. New collaboration and networking between SA and Europe are envisaged with the prospects of including other clinical sites in the Eastern Cape, Kwazulu-Natal and Limpopo provinces of South Africa, Uganda and Ethiopia for a phase III trial (in future). Building of research capacity in more neglected regions and countries may only benefit from this initiative when a larger phase III trial is launched. However, the project will strengthen current competencies in the institution.
- The proposal might not completely fit the mission and objectives of EDCTP. The main aim is to seek support for a proof-of-concept clinical trial, in preparation for a larger phase 3 trial. The ultimate intention is to improve the drug treatment of patients with HIV and TB co-infection; both are infectious diseases that have been prioritised by the EDCTP.
- The proposal is important and has considerable potential merit and the budget request is realistic.

European & Developing Countries Clinical Trials Partnership
P.O. Box 93015, 2509 AA ▪ Laan van Nieuw Oost Indië 334, 2593 CE ▪ The Hague, The Netherlands
Tel +31 (0)70-3440880 ▪ Fax +31 (0)70-3440899 ▪ Email info@edctp.org ▪ Web www.edctp.org

EDCTP-EEIG is registered in the Hague, The Netherlands. Chamber of Commerce file no. 27259980

EDCTP

Reviewer 3:

- Excellent team well balanced. Only one applicant is a woman. No concerns about ability to do the work.
- TB-IRIS is a substantial problem causing additional morbidity and mortality for patients and burden for the health service. The proposed study is probably the most important study that needs to be conducted for prevention of TB-IRIS. The proposal will almost certainly deliver important insights, both for prevention of TB-IRIS and in improving our understanding of TBIRIS.
- Very well designed study. Time frame and budget are both reasonable. No ethical/safety concerns that have not been addressed.
- If the study is successful, it will almost certainly lead to a phase III trial which the group are very well placed to lead.
- If it is unsuccessful it will still collect a lot of important data that will improve our understanding of TB-IRIS and help establish the way forward.
- Fits very well, only issue is that this is a treatment trial for the management of TB-IRIS and so may not be core to the aims of EDCTP.
- As mentioned above, this is a very important trial, with a great team, that is very likely to lead to new trials and to have a real public health impact. Only question is whether prevention of TB-IRIS is a core aim of EDCTP.

European & Developing Countries Clinical Trials Partnership
P.O. Box 93015, 2509 AA ▪ Laan van Nieuw Oost Indië 334, 2593 CE ▪ The Hague, The Netherlands
Tel +31 (0)70-3440880 ▪ Fax +31 (0)70-3440899 ▪ Email info@edctp.org ▪ Web www.edctp.org

EDCTP-EEIG is registered in the Hague, The Netherlands. Chamber of Commerce file no. 27259980
